# Supplementary material for: Functional Downregulation of PD-L1 and PD-L2 by CpG and non-CpG Oligonucleotides in Melanoma Cells
Source: Cancers (Basel). 2022 Sep 27;14(19):4698. doi: 10.3390/cancers14194698 (PMC9562717; doi:10.3390/cancers14194698)
Supplement: Supplementary file 1 [file cancers-14-04698-s001.zip › cancers-1787874-supplementary.pptx]

## Slide 1
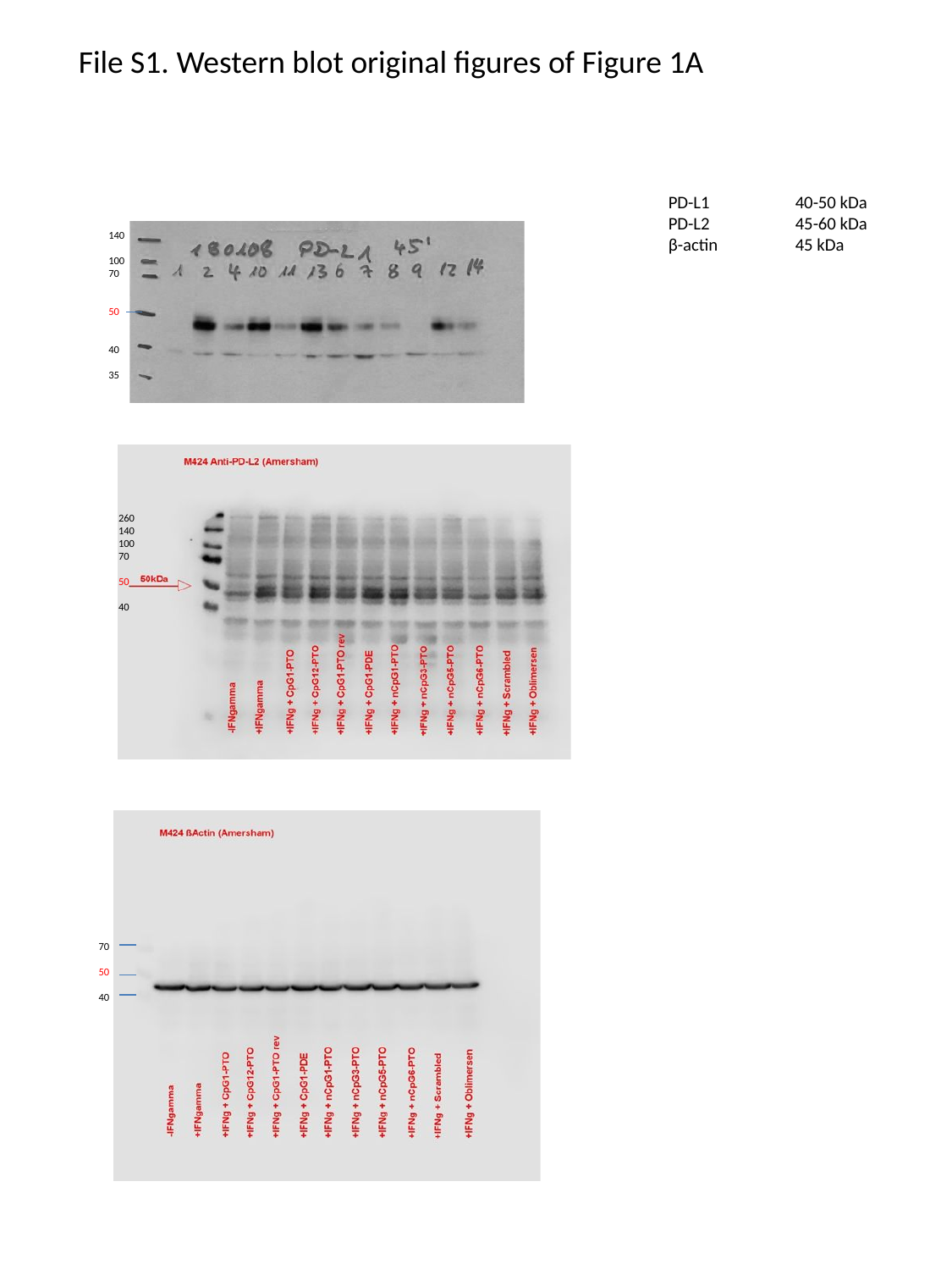

File S1. Western blot original figures of Figure 1A
PD-L1	40-50 kDa
PD-L2	45-60 kDa
β-actin	45 kDa
140
100
70
50
40
35
260
140
100
70
50
40
70
50
40

## Slide 2
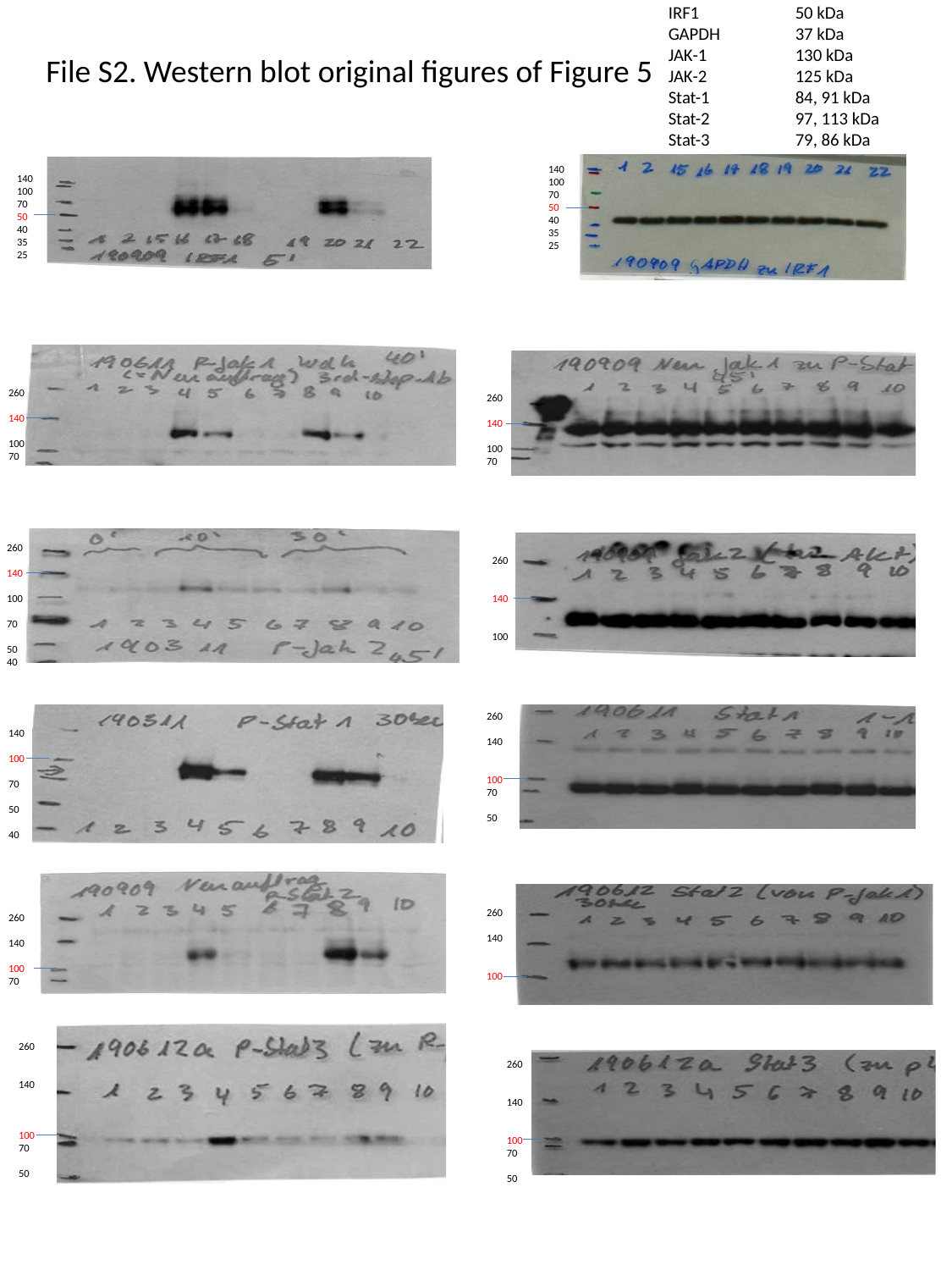

IRF1	50 kDa
GAPDH	37 kDa
JAK-1	130 kDa
JAK-2	125 kDa
Stat-1	84, 91 kDa
Stat-2	97, 113 kDa
Stat-3	79, 86 kDa
File S2. Western blot original figures of Figure 5
140
100
70
50
40
35
25
140
100
70
50
40
35
25
260
140
100
70
260
140
100
70
260
140
100
70
50
40
260
140
100
260
140
100
70
50
140
100
70
50
40
260
140
100
260
140
100
70
260
140
100
70
50
260
140
100
70
50

## Slide 3
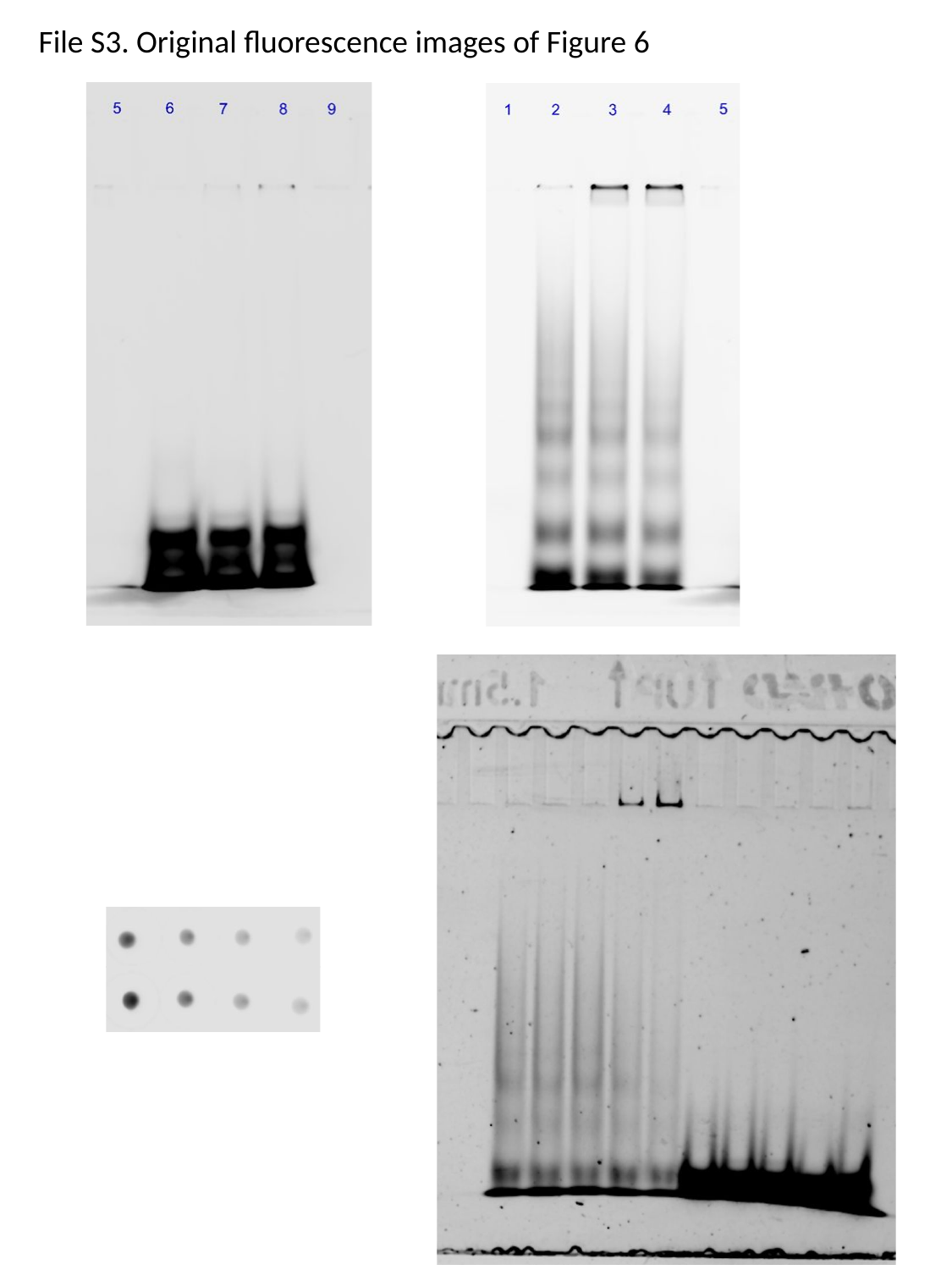

File S3. Original fluorescence images of Figure 6
