# Supplementary figures and images for: Functional Downregulation of PD-L1 and PD-L2 by CpG and non-CpG Oligonucleotides in Melanoma Cells
Source: Cancers (Basel). 2022 Sep 27;14(19):4698. doi: 10.3390/cancers14194698 (PMC9562717; doi:10.3390/cancers14194698)

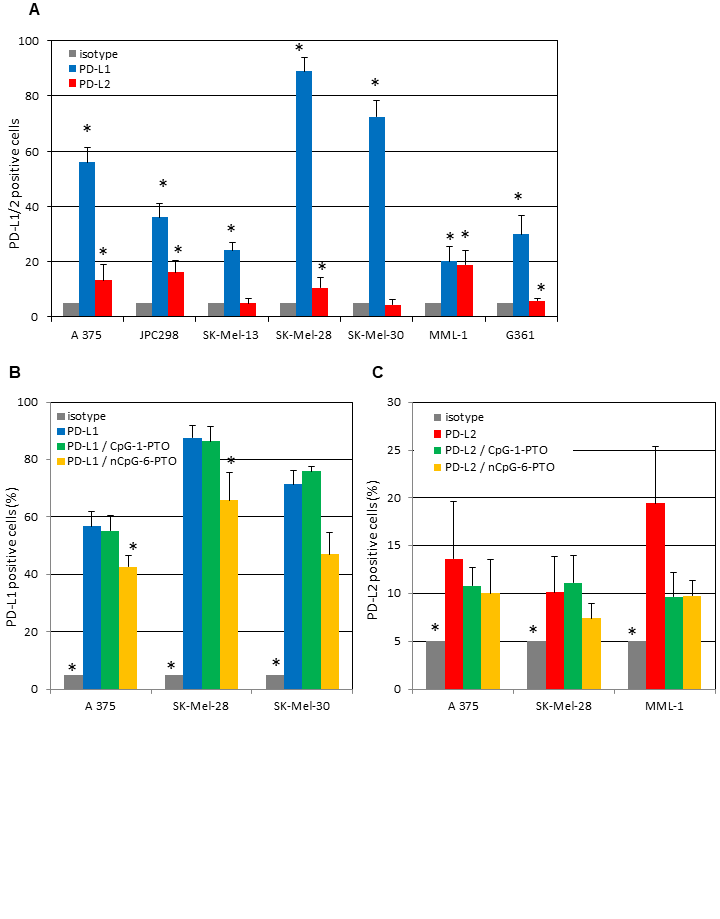

Supplement: Supplementary file 1 [file cancers-14-04698-s001.zip › Figure S1.TIF]

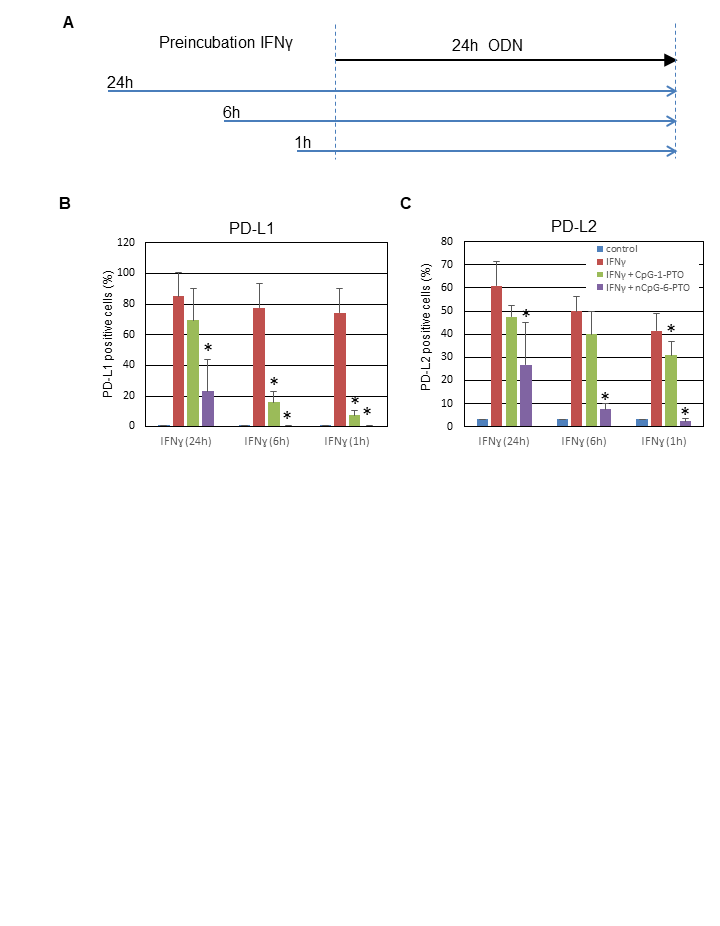

Supplement: Supplementary file 1 [file cancers-14-04698-s001.zip › Figure S2.TIF]

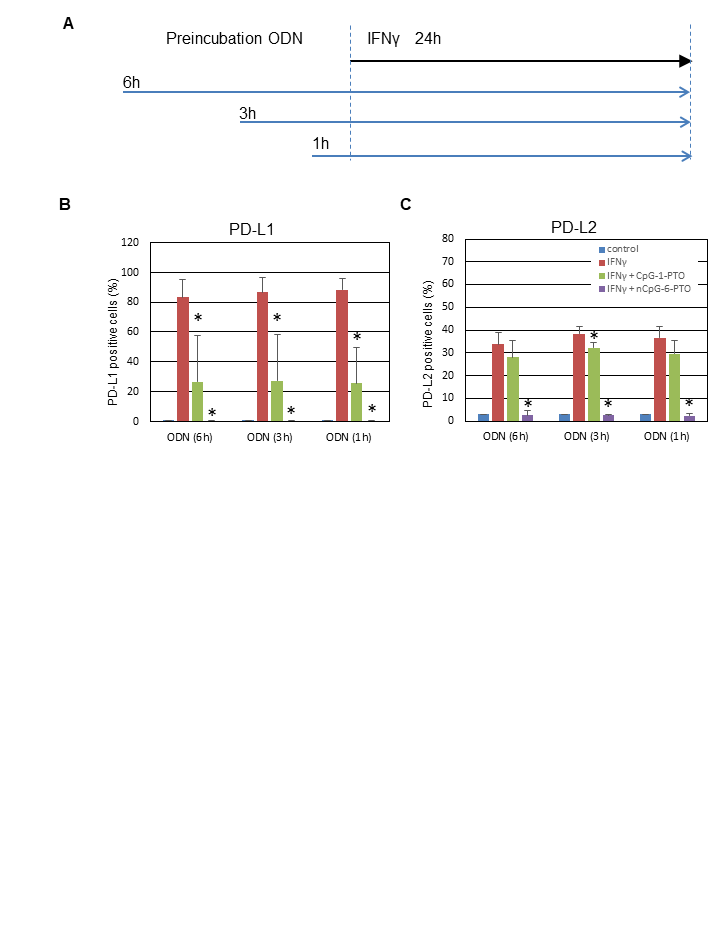

Supplement: Supplementary file 1 [file cancers-14-04698-s001.zip › Figure S3.TIF]

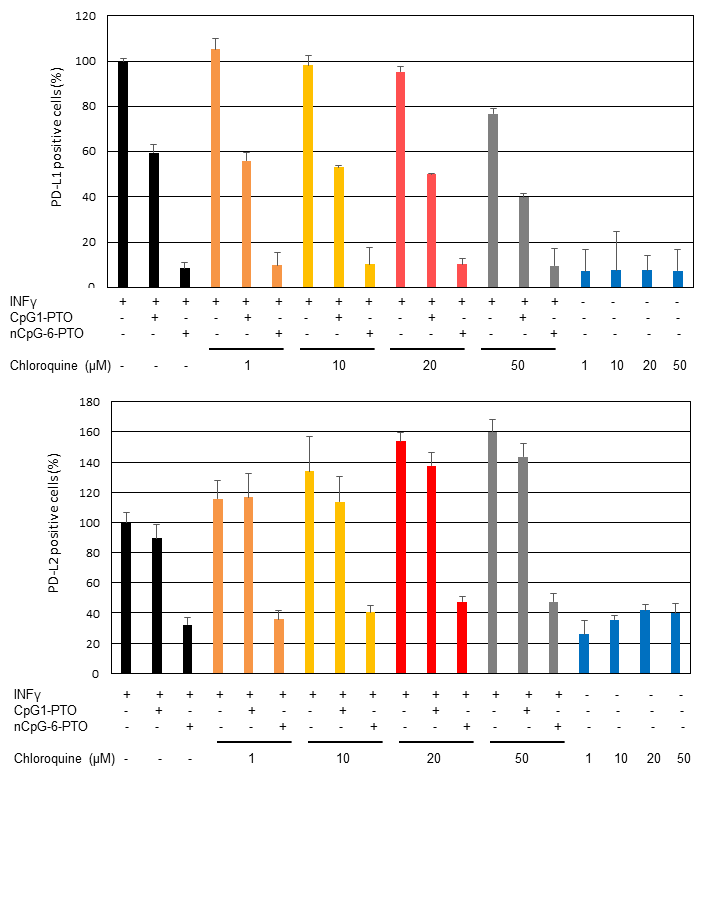

Supplement: Supplementary file 1 [file cancers-14-04698-s001.zip › Figure S4.TIF]

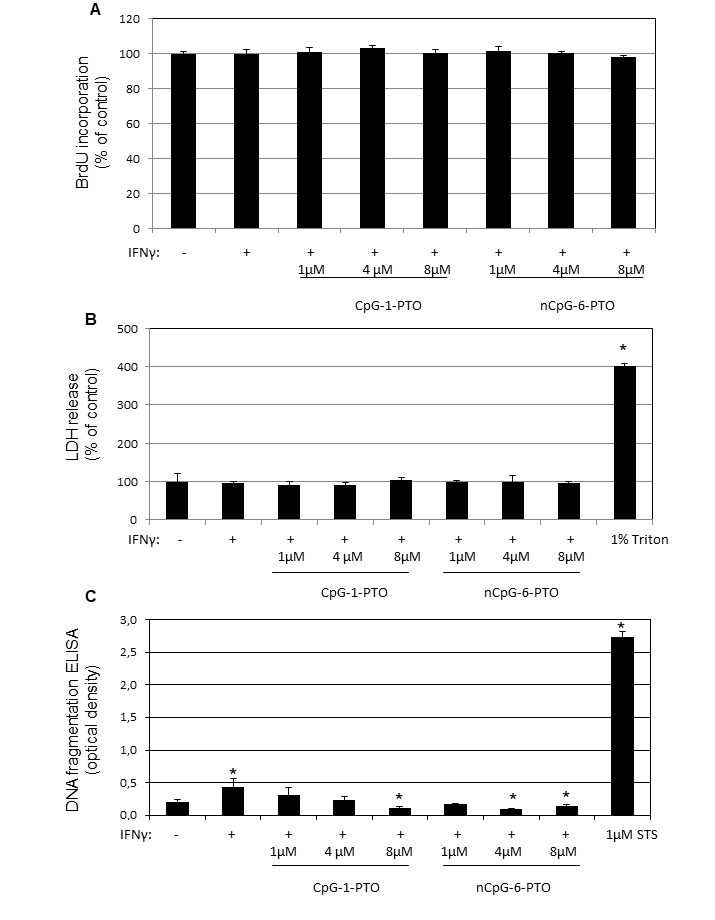

Supplement: Supplementary file 1 [file cancers-14-04698-s001.zip › Figure S5.TIF]
